# Increased Paracetamol Bioavailability after Sleeve Gastrectomy: A Crossover Pre- vs. Post-Operative Clinical Trial

**DOI:** 10.3390/jcm8111949

**Published:** 2019-11-12

**Authors:** Daniel Porat, Milica Markovic, Moran Zur, Noa Fine-Shamir, Carmil Azran, Gad Shaked, David Czeiger, Julie Vaynshtein, Ilya Replyanski, Gilbert Sebbag, Arik Dahan

**Affiliations:** 1 Department of Clinical Pharmacology, School of Pharmacy, Faculty of Health Sciences, Ben-Gurion University of the Negev, Beer-Sheva 84105, Israel; porat86@gmail.com (D.P.); milica@post.bgu.ac.il (M.M.); moranfa@post.bgu.ac.il (M.Z.); fineno@post.bgu.ac.il (N.F.-S.); 2 Clinical Pharmacy, Herzliya Medical Center, Herzliya 46140, Israel; CarmilA@hmc.co.il; 3 Department of Surgery B, Soroka University Medical Center, Beer Sheva 84101, Israel; gadishndani@gmail.com (G.S.); czeiger@bgu.ac.il (D.C.); julie.vaynshtein@gmail.com (J.V.); IlyaRe@clalit.org.il (I.R.); GilbertS@clalit.org.il (G.S.)

**Keywords:** paracetamol (acetaminophen), bariatric surgery, laparoscopic sleeve gastrectomy, drug absorption, glucuronidation, gastric emptying

## Abstract

Oral drug bioavailability may be significantly altered after laparoscopic sleeve gastrectomy (LSG), the most popular bariatric procedure worldwide. Paracetamol (acetaminophen) is the post-bariatric analgesic/antipyretic drug of choice. In this work we studied and analyzed the LSG effects on systemic bioavailability and pharmacokinetics of paracetamol after oral administration of solid vs. liquid dosage form. A 4-armed, pharmacokinetic, crossover trial was performed in patients enrolled for LSG. Single paracetamol dose (500 mg), as caplet (*n* = 7) or syrup (*n* = 5), was administered before vs. 4–6 months post-LSG. Bioavailability was enhanced after LSG; in the caplet groups, average AUC_0–t_ increased from 9.1 to 18.6 µg·h/mL with AUC_0–t_ difference of 9.5 µg·h/mL (95% CI 4.6–14.5, *p* = 0.003). C_max_ increased from 1.8 (95% CI 1.2–2.5) to 4.2 µg/mL (3.6–4.8) after LSG (*p* = 0.032). In the syrup groups, AUC_0–t_ increased from 13.4 to 25.6 µg·h/mL, with AUC_0–t_ difference of 12.2 µg·h/mL (95% CI 0.9–23.5, *p* = 0.049). C_max_ changed from 5.4 (95% CI 2.5–8.4) to 7.8 µg/mL (6.1–9.6), and systemic bioavailability was complete (102%) after the surgery. Overall, decreased paracetamol exposure in obesity, with recovery to normal drug levels (caplet) or even higher (syrup) post-LSG, was revealed. In conclusion, attention to paracetamol effectiveness/safety in obesity, and after bariatric surgery, is prudent.

## 1. Introduction

Treating obesity is challenging, given the limited pharmacological options available and the difficulty in maintaining long-term lifestyle interventions [1]. Bariatric surgery is the mainstay of treatment for morbid obesity, capable of preserving long-term weight loss [2,3,4]. Bariatric surgery techniques include: (1) gastric banding, placing an adjustable band around the top part of the stomach; (2) gastric bypass surgery, including Roux-en-Y gastric bypass (RYGB), which is the creation of a small gastric pouch, connected to a limb of the small intestine bypassing the larger part of the stomach and proximal gut, and the more recent single-anastomosis gastric bypass (or mini-gastric bypass) in which the upper part of the stomach is divided into a tube and then joined to a loop of intestine; and (3) sleeve gastrectomy, in which a large portion of the stomach is removed by a longitudinal resection along the greater curvature. Being minimally invasive and not inferior to gastric bypass [5,6], laparoscopic sleeve gastrectomy (LSG) has become the most common procedure worldwide, including in the United States and Europe [7,8,9].

The modified GI anatomy after bariatric surgery may significantly affect the oral absorption of many drugs [10,11]. Although malabsorptive bypass procedures are highly prone to cause such effects, restrictive surgeries, e.g., LSG, may also alter the pharmacokinetics (PK) of various drugs [12,13,14]. Many parameters that dictate the absorption of a drug may be altered after the surgery, e.g., stomach volume and pH, gastric motility and transit time [10,15]. The limited literature and insufficient awareness of drug absorption after bariatric surgery may deprive necessary drug/dosage adjustments; thus, uncovering the surgery’s effects on drug therapy should allow better patient care.

Solid immediate-release oral dosage forms (e.g., tablets, caplets) must first disintegrate, and consequent drug dissolution/solubilization will allow the drug to permeate into the GI membrane and be absorbed. In liquid dosage forms (e.g., syrup), on the other hand, the drug is already dissolved, and since LSG can significantly alter these processes, differences between solid and liquid dosage forms may be expected after the surgery [16].

In this work, we have studied the systemic bioavailability and pharmacokinetics of paracetamol from caplet and syrup dosage forms, before vs. after LSG, hypothesizing altered plasma drug concentrations after LSG. Paracetamol (acetaminophen) is a very common antipyretic and analgesic agent. It is well absorbed after oral administration, with bioavailability of 70–90% attributable to first pass metabolism [17,18,19]. Paracetamol is also the analgesic/antipyretic drug of choice for bariatric patients, who should avoid non-steroidal anti-inflammatory drugs (NSAIDs) due to increased risk of bleeding and ulcerations.

A clinical, 4-armed, crossover, pharmacokinetic trial was designed and performed in patients with morbid obesity enrolled for LSG. Single oral paracetamol dose (500 mg) in a caplet vs. syrup dosage form was administered, before vs. 4–6 months after LSG. A thorough mechanistic analysis of the obtained pharmacokinetics is provided, with potential applicability to other drugs and bariatric procedures as well. Altogether, the data revealed in this work may allow better drug therapy and overall patient care after bariatric surgery.

## 2. Methods

### 2.1. Subjects

Nine patients with morbid obesity (body mass index (BMI) > 40 kg/m^2^) were recruited to the study. All patients were planned to undergo a sleeve gastrectomy operation in the Soroka University Medical Center, Department of Surgery B. Patients were not recruited if they participated in previous clinical trials, if they had previous bariatric surgery, renal/hepatic impairment, paracetamol hypersensitivity or were pregnant/breastfeeding.

### 2.2. Experimental Design

The study population was patients with morbid obesity enrolled for LSG. There were four study groups: paracetamol caplet (Acamol^®^, Teva Pharmaceutical Industries Ltd., Petah-Tikva, Israel) before vs. after LSG, and paracetamol syrup (Acamoli Forte^®^, 250 mg/5 mL, Teva Pharmaceutical Industries Ltd., Petah-Tikva, Israel) before vs. after LSG. In this clinical study, patients were administrated a 500 mg paracetamol caplet or syrup randomly, followed by blood samples withdrawal at set times (0, 15, 30, 45 and 60 min, 1.5, 2, 2.5, 3, 3.5, 4, 5, 6, 7, 8, 10 and 12 h) after drug ingestion. After a washout period of one to two weeks, the PK study was repeated for the same patient with the other dosage form. The same protocol was repeated 4–6 months after the surgery, with each patient serving as their own control, increasing the study’s statistical power. All 7 patients who got a caplet, and 4 of the 5 patients who got syrup before LSG, also participated in the post-LSG caplet or syrup groups, respectively. We aimed for each patient to participate in all four study groups, and 3 of the 9 patients did. The study protocol was approved by the institutional review board of Ben-Gurion University School of Medicine (institutional board request number 0302-15-SOR) and written informed consent was obtained from all participants. Since we studied the effects of a medical condition, sleeve gastrectomy, on the exposure of paracetamol, with no interventional trial or new treatment, registration of this non-interventional clinical study was not needed.

### 2.3. Quantification of Paracetamol Plasma Concentrations

Plasma samples were analyzed for paracetamol content by ultra-performance liquid chromatography (Waters Acquity UPLC H-Class system equipped with PDA detector and controlled by Empower software), using a previously reported method with minor modifications [20]. Blood samples were collected, centrifuged (5000 rpm for 10 min), and the plasma was assayed for drug content; 200 µL plasma sample was mixed with 20 µL of 35% perchloric acid, vortexed for 1 min and centrifuged at 14K rpm for 10 min. Supernatant was then filtered, and 80 µL was injected to the UPLC.

Analysis was done on a Waters (Milford, MA) Xterra UPLC RP18 3.5 μm 4.6 × 250 mm column, with a gradient mobile phase of 90:10 going to 15:85 (*v*/*v*) distilled water:acetonitrile at a flow rate of 1 mL/min. Total run time was 8 min, while paracetamol retention time was 5.1 min, with a detection wavelength of 245 nm. The calibration curve was linear in the range of 0.25–10 µg/mL. Both inter- and intraday coefficients of variation were smaller than 1%.

### 2.4. Pharmacokinetic Analysis

Paracetamol plasma concentrations were used to create PK profiles by plotting drug concentration vs. time curves. PK parameters were determined using PK Solver 2.0 software. Non-compartmental PK parameters including maximum plasma concentration (C_max_), time to C_max_ (T_max_), area under the concentration-time curve from time zero to 12 h (AUC_0–t_), relative clearance (CL/F) and volume of distribution (Vd/F) after oral administration, and the drug’s half-life (t_½_) in plasma were calculated. Systemic oral bioavailability of paracetamol was determined as the relative area under the curve (AUC) of the drug to an average AUC value (adjusted to dose) of intravenous (IV) paracetamol administrated (taken from literature) [21]. The researcher and data analyst were blinded during the entire course of plasma quantification and pharmacokinetic analysis.

### 2.5. Statistical Analysis

Table 1 values are expressed as mean ± standard deviation (in parenthesis); pharmacokinetic values are expressed as mean ± standard error (in parenthesis). Results were statistically analyzed using a two tailed paired *t*-test; *p* < 0.05 was termed statistically significant.

## 3. Results

### 3.1. Baseline Characteristics

All patients had significant decrease in weight and BMI at 4–6 months after LSG; mean BMI decreased from 43.8 to 34.6 kg/m^2^ (9.2 kg/m^2^ difference, 95% CI 7.1 to 11.3, *p* < 0.001) and average weight decreased from 125 to 99 kg (26 kg difference, 95% CI 18 to 31, *p* < 0.001). All other baseline characteristics (average systolic and diastolic blood pressure, average heart rate, and smoking status) were unchanged after LSG relative to before (Table 1).

### 3.2. Paracetamol Caplets

**Figure 1:**
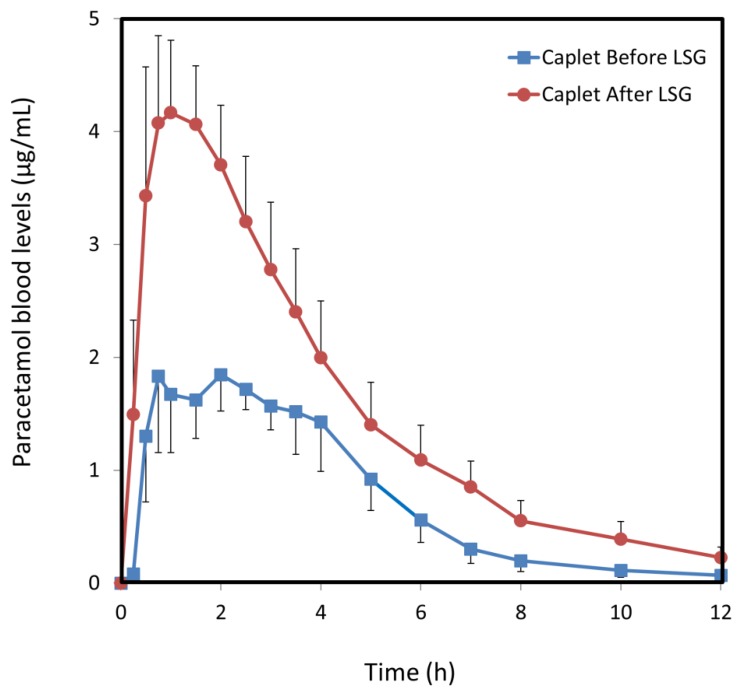
Paracetamol blood levels following oral administration of a 500 mg paracetamol caplet. The study was carried out a few weeks before (blue squares) vs. 4–6 months after laparoscopic sleeve gastrectomy (LSG) (red circles). Data are presented as mean ± SE; *n* = 7. LSG, laparoscopic sleeve gastrectomy.

Paracetamol plasma profiles from a caplet dosage form, before vs. after LSG are presented in Figure 1. The systemic bioavailability of the drug was significantly higher in the post-surgery group. Furthermore, all seven patients participating in both the pre- and post-surgery caplet groups had increased AUC_0–t_ after LSG. The comparison of pharmacokinetic parameters between the pre- and post-surgery groups is presented in Table 2; doubled systemic bioavailability in the post-surgery group (AUC_0–t_ enhancement of 104%), and 133% higher C_max_ were obtained. Average AUC_0–t_ was increased from 9.1 to 18.6 µg·h/mL. AUC_0–t_ difference was 9.5 µg·h/mL (95% CI 4.6 to 14.5, *p* = 0.003). C_max_ increased from 1.8 (95% CI 1.2 to 2.5) to 4.2 µg/mL (3.6 to 4.8, *p* = 0.032) and CL/F decreased from 57 (95% CI 30 to 84) to 32 (95% CI 14 to 51) (*p* = 0.004) after LSG.

### 3.3. Paracetamol Syrup

**Figure 2:**
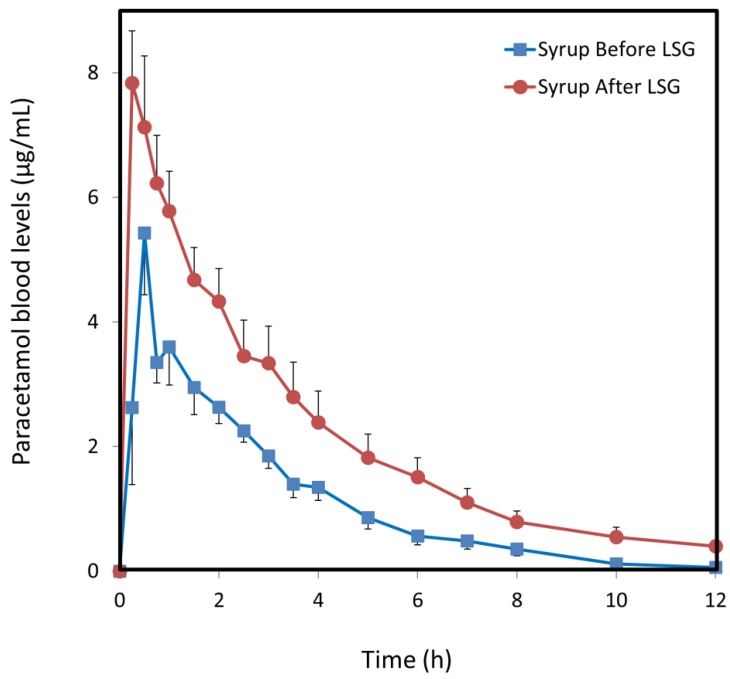
Blood levels following oral administration of 500 mg paracetamol syrup. The study was carried out a few weeks before (blue squares) vs. 4–6 months after LSG (red circles). Data are presented as mean ± SE; *n* = 4–5.

Paracetamol plasma profiles from a syrup dosage form before vs. after LSG are presented in Figure 2. Once again, higher plasma drug concentrations were achieved in the post-surgery group, with a 91% higher AUC_0–t_ compared to pre-surgery (Table 2). AUC_0–t_ was increased from 13.4 to 25.6 µg·h/mL, with AUC_0–t_ difference of 12.2 µg·h/mL (95% CI 0.9 to 23.5, *p* = 0.049) and CL/F decreased from 35.3 (95% CI 21.8 to 48.8) to 21.0 (95% CI 7.3 to 34.8, *p* = 0.008) after LSG. C_max_ changed from 5.4 (95% CI 2.5 to 8.4) to 7.8 µg/mL (6.1 to 9.6). As in the caplet groups, this increased AUC was evident in all individual patients. In the post-surgery syrup group, the entire dose reached the blood as can be seen from the complete bioavailability (Table 2).

Combining the results from both the caplet and syrup groups, the elimination half-life (t_½_) was longer after LSG; t_½_ differences were not statistically different after LSG in the caplet or syrup groups individually.

Comparing the results between the dosage forms, a general trend of enhanced drug exposure is witnessed in the syrup groups relative to the caplet groups (both before and after LSG). However, the differences in AUC and C_max_ between the syrup and caplet groups, both before and after surgery, were not statistically significant. T_max_ had a general trend of being shorter from syrup vs. caplet, correlating with faster gastric emptying of liquid vs. solid content [22]. Furthermore, no difference in systemic bioavailability was observed between males and females, in either dosage form.

## 4. Discussion

Oral drug bioavailability may be significantly altered after bariatric surgery because of the modified GI anatomy. Parameters such as drug solubility/dissolution, permeability and metabolism may all be affected by the bariatric procedure. Scientific rationale mainly supports decreased or unchanged drug absorption after bariatric surgery, and indeed, lower post-operative oral bioavailability was reported for thyrosine kinase inhibitors, antidepressants, immunosuppressants and other drugs such as propranolol and hydrochlorothiazide [23,24,25]. Meanwhile, reports of increased drug exposure after the surgery are less common. In this study, we have shown that oral bioavailability of paracetamol is doubled after LSG. The clinical implications of these unexpected results relate to both efficacy and toxicity of the drug; specifically, for paracetamol, caution should be used as higher drug exposure can increase risk of hepatotoxicity at maximal daily doses, and dose adjustment should be considered.

Paracetamol has systemic oral bioavailability of 70–90% in the general population [17,18,19]. We found that the oral bioavailability of paracetamol in patients with obesity is significantly lower (~50%); in the same patients after LSG, bioavailability was recovered to the normal levels (in the case of solid dosage form), and even more than that, with 100% bioavailability for liquid dosage form.

After LSG, only ~20% of the original gastric volume remains, and acid secretion from the stomach is decreased. This may severely hamper the solubility/dissolution of marginally soluble drugs, which require adequate gastric volume in order to fully dissolve the entire drug dose [26]. However, paracetamol is a high solubility drug, that is equally soluble in the entire physiologic pH range, with experimental aqueous solubility of 23.7 mg/mL (at 37 °C) [27]. Hence, in order to dissolve a 500 mg dose, a volume of 21 mL of water is required, which is available even after LSG. In other words, the limited volume of the gastric pouch (~50 mL) is still sufficient to fully dissolve the drug dose and not affect the absorption of 500 mg paracetamol. This analysis clarifies why paracetamol bioavailability is not expected to decrease after LSG; however, the increased (rather than unchanged) bioavailability revealed in this study still remains to be explained.

Paracetamol undergoes phase II metabolism in the liver and the intestinal wall to form inactive glucuronide and sulphate metabolites. These mechanisms of metabolism also occur pre-systemically, resulting in the incomplete bioavailability of the drug [28,29]. Paracetamol primarily undergoes glucuronidation (about half the dose) facilitated by enzymes of the uridine 5’-diphospho-glucuronosyltransferase (UGT) family, with UGT1A9 being the predominant isoform in the liver and UGT1A10 in the gut [30]. Another major metabolism process of paracetamol is sulfate conjugation (about a third of the dose) mediated by the sulfotransferase enzymes: SULT1A1, SULT1A3/1A4 and SULT1E1 [31]. A third, minor but important metabolic pathway of this drug is CYP2E1-mediated oxidation to the toxic n-acetyl-p-benzoquinone imine (NAPQI) metabolite [32].

Glucuronidation was reported to be enhanced in individuals with morbid obesity [33]. Sorrow et al. showed that children with obesity were more likely to have elevated levels of glucuronide and sulfate metabolites of paracetamol, using metabolomics profiling [34]. Abernethy et al. analyzed the clearance of paracetamol, as well as other UGT substrates, concluding that glucuronidation capacity increases in proportion to total body weight [35,36]. This higher metabolism can explain the lower exposure of paracetamol in the pre-surgery arms of this study.

As for the post-surgery arms, patients undergoing LSG experience great weight loss, which was also the case here (Table 1). Weight loss is accompanied by the loss of adipose tissue, which is rich in glucuronide enzymes, and by reduced liver size. As a result, LSG is expected to decrease the extent of glucuronidation, leading to higher post-surgery paracetamol plasma levels, which finds corroboration in our results. In fact, after LSG, paracetamol levels from syrup were even greater than the levels in the general population (Table 2). In other words, following LSG-induced weight loss, our results suggest a ’rebound’ effect, potentially due to decreased enzymatic expression, leading to lower levels of paracetamol metabolism than in the general population.

Independently of weight loss, LSG has been shown to significantly accelerate gastric emptying [37,38]; the remaining post-surgery stomach pouch cannot withhold the drug dose for as long as the non-operated stomach does [39]. Gastric emptying is the rate-limiting step of paracetamol absorption, determining the drug’s T_max_ [40,41]. As a result, after LSG, the entire drug dose quickly reaches the duodenum at once, potentially saturating UGT, thereby escaping pre-systemic glucuronidation, resulting in higher paracetamol systemic bioavailability. Indeed, intestinal wall UGT enzymes were shown to be saturated rapidly by paracetamol in rats [42].

The analysis involving gastric emptying is relevant to the syrup dosage form, in which the drug is already dissolved and ready for absorption. However, it is less relevant in the caplet group, since disintegration/dissolution has to occur prior to absorption. Indeed, T_max_ revealed in our study supports this analysis: after surgery, T_max_ was 60 min in the caplet group vs. only 15 min in the syrup group. Overall, our finding of complete paracetamol absorption accompanied by lower CL/F value (Table 2) after LSG correlates well with these mechanisms of enhanced glucuronidation in obesity and decreased glucuronidation after LSG.

Additionally, a double-peak phenomenon was evident in the pre-LSG caplet and syrup groups, both in the individual patients and in the average curves, but not after the surgery (Figure 1 and Figure 2). Prior to surgery, gastric emptying is delayed as described above, when some of the drug is held in the stomach after the rest of the dose has been absorbed. The second peak in the plasma drug concentration profiles is attributable to this pre-LSG delay in gastric emptying. After the surgery, this two phase gastric emptying is eliminated, and the double peak phenomenon disappears.

It should be noted that systemic oral bioavailability (F) values were calculated relative to literature paracetamol AUC after IV administration to healthy volunteers [21]; however, using IV data from subjects with obesity [43] resulted in similar F values.

Our results, revealed for LSG, are also relevant for other bariatric procedures. In gastric bypass, the UGT1A10-rich duodenum [44] is bypassed, potentially leading to even less post-operative first-pass metabolism and further enhanced systemic bioavailability of paracetamol. Therefore, we can predict a similar trend after gastric bypass. On the other hand, reports of higher paracetamol exposure after gastric bypass would not necessarily suggest higher drug levels after LSG, because bypass surgeries involve more supporting factors than sleeve does.

Other drugs that undergo extensive glucuronidation are expected to show similar results. Another important analgesic undergoing glucuronidation as a major metabolic pathway is morphine. Lloret-Linares et al. studied the PK of morphine and its glucuronide metabolites in patients with obesity and 6 months after RYGB, arguing that glucuronidation extent decreased significantly with post-RYGB weight loss [45]. The benzodiazepine agents lorazepam and oxazepam were also shown to have enhanced glucuronidation in obesity [36]. Additionally, other important medications that are significantly eliminated by glucuronidation may exhibit reduced effectiveness in obesity, including lamotrigine [46], olanzapine [47], raloxifene [48], dapagliflozin [49] and others, emphasizing the clinical relevance of the analysis presented in this article. Interestingly, while most glucuronide metabolites are inactive, for some drugs, such as ezetimibe [50,51], the glucuronide metabolite is also active and even more potent that the parent drug, so these agents may actually be less effective after LSG. Other mechanisms potentially involved in reduced drug exposure and effect after bariatric surgery, in addition to altered metabolism and gastric volume and pH, are decreased gastrointestinal motility, hampered passive and carrier-mediated permeability, delayed bile secretion and even decreased food intake, which may limit the absorption of certain drugs taken after a meal [10].

In summary, this paper covered the effects of obesity and LSG on various mechanisms involved in the exposure of orally administered drugs. A limitation of this study was the relatively small number of participants; however, the results showed clear statistical significance. Strengths include the cross-over design, and the thorough mechanistic analysis of the results.

Overall, changes of drug metabolism after oral administration may be expected among patients with obesity. Then, bariatric surgery may reverse these obesity-related metabolic changes. Therefore, while LSG may decrease the solubility/dissolution of drugs with marginal solubility, it may simultaneously decrease drug metabolism (e.g., glucuronidation), resulting in decreased, increased or unchanged, and overall unpredictable, drug exposure. Limited data are currently available on the effects of obesity on drug disposition, and even less is known on the effects of bariatric surgeries, including LSG. Further research in this field is therefore highly needed. In the meantime, applying measures such as monitoring drug levels and symptoms in patients after bariatric surgery, and consulting with a clinical pharmacist regarding drug administration in these cases is prudent and strongly advised.

## 5. Conclusions

Compared to healthy individuals, paracetamol plasma levels are significantly decreased in patients with obesity. After LSG, drug exposure increases, and AUC following caplet ingestion is comparable to the values of healthy subjects. Paracetamol syrup after surgery allows complete systemic bioavailability. Caution should be used as higher paracetamol exposure can increase risk of hepatotoxicity at maximal daily doses, and dose adjustment should be considered. Given these results and the limited knowledge on post-LSG pharmacotherapy, further research is encouraged.

## Figures and Tables

**Table 1:** Baseline patient characteristics, before vs. after laparoscopic sleeve gastrectomy (LSG). SBP, systolic blood pressure; DBP, diastolic blood pressure; HR, heart rate. * *p* < 0.001.

Parameter	Pre-LSG	Post-LSG
**Age (years)**	38.9 (13.6)	
**Females**	6	
**Males**	3	
**Smokers**	4	
**Height (cm)**	167 (9)	
**Weight (kg)**	125 (17)	99 (19) *
**BMI (kg/m^2^)**	43.8 (4.0)	34.6 (4.6) *
**SBP (mmHg)**	135 (24)	133 (20)
**DBP (mmHg)**	74 (17)	78 (15)
**HR (bpm)**	85 (18)	81 (19)

**Table 2:** Pharmacokinetic parameters of the four study groups. F (systemic bioavailability) was calculated from oral AUC relative to literature paracetamol AUC after IV administration [21]. * *p* < 0.05, and ** *p* < 0.01.

	Caplet		Syrup	
	Before	After	Before	After
**N**	7	7	5	4
**t_½_ (h)**	2.1 (0.7)	2.4 (0.4)	1.9 (0.3)	2.7 (0.3)
**T_max_ (h)**	0.75	1	0.5	0.25
**C_max_ (µg/mL)**	1.8 (0.7)	4.2 (0.6) *	5.4 (1.0)	7.8 (0.9)
**AUC_0–t_ (µg·h/mL)**	9.1 (1.3)	18.6 (3.2) **	13.4 (1.5)	25.6 (5.0) *
**F (%)**	36 (5.3)	74 (13.0) **	54 (6.3)	102 (20.3) *
**CL/F (L/h)**	57 (10.9)	32 (7.4) **	37 (3.2)	22 (3.4) **
**Vd/F (L)**	53 (15.5)	75 (12.0)	148 (43)	101 (16.2)
